# Rheumatoid factor as predictor of response to treatment with anti-TNF alpha drugs in patients with rheumatoid arthritis: Results of a cohort study

**DOI:** 10.1097/MD.0000000000014181

**Published:** 2019-02-01

**Authors:** Pedro Santos-Moreno, Guillermo Sánchez, Carlos Castro

**Affiliations:** a Biomab IPS: Rheumatoid Arthritis Center; b Fundación Universitaria de Ciencias de la Salud, Clinical Epidemiology; c SIIES Research and Education in Health, Fundación Universitaria de Ciencias de la Salud, Bogotá, Colombia.

**Keywords:** anti-cyclic citrullinated peptide antibodies, disability, remission, rheumatoid arthritis, rheumatoid factor

## Abstract

We determined whether rheumatoid factor (RF) and anti-cyclic citrullinated peptide antibody (ACPA) can predict remission or severe disability in rheumatoid arthritis (RA) patients treated with anti-tumor necrosis factor (TNF) alpha drugs.

We performed a cohort study based on the clinical data from a referral center for the treatment of RA in Bogotá, Colombia, were included patients aged ≥18 years with diagnosis of RA with an active disease and for whom a treatment scheme was begun with anti-TNF alpha medication, with a minimum follow-up time of 12 months. Disease activity of Rheumatoid Arthritis was assessed through measurement of RF, ACPA, disease activity score (DAS28), and health assessment questionnaire (HAQ). We calculated the incidence rates (IRs) for remission and severe disability. We also calculated the incidence rate ratio (IRR) for each outcome by adjusting for possible confounders using the Poisson regression method. The hypothesis was tested with a *P* value of <.05. Statistical analysis was performed in Stata 15.

We included 400 patients receiving an anti-TNF alpha agent. Median age was 60 years, and 322 patients were women (80.5%). RF was positive in 357 patients (89%), ACPA in 348 patients (87%), and co-positivity in 324 patients (81%). Median follow-up was 41 months (range, 12–79 months). The IR for remission was 23 per 100 person-years in RF-negative patients and 16 per 100 person-years in RF-positive patients. The adjusted IRR (age sex, treatment, and ACPA) was 1.51 (95%CI, 1.05–2.18). The IR for severe disability was 10.8 per 100 person-years in the RF-positive cohort and 2.3 per 100 person-years in the RF-negative cohort. The IRR adjusted for these factors was 4.37 (95%CI, 1.6–12). Co-positivity had a similar behavior to RF. No differences were recorded in the rates of remission or disability in ACPA-positive and ACPA-negative patients.

Our findings suggest that remission is less frequent and severe disability more frequent in RF-positive patients treated with anti-TNF alpha agents than in RF-negative patients.

**Key Points**

- Anti-TNF alpha agents are expensive and can cause adverse effects; therefore, it is necessary to identify predictors of an adequate response to treatment with these drugs.
- Rheumatoid factor is a useful marker for predicting response to therapy with anti-TNF agents.
- The remission rate for RF-negative patients was higher than that of RF-positive patients.
- The rate of severe disability was higher in RF-positive patients than in RF-negative patients.

## 1 Introduction

Rheumatoid arthritis (RA) is a chronic inflammatory autoimmune disease of unknown etiology that is characterized by polyarticular pain and swelling.^[1,2]^ RA should be diagnosed and treated early in order to limit joint damage, associated complications, and progressive disability.^[3]^ The worldwide prevalence of RA is estimated to be around 1%, with considerable variation depending on the population studied.^[4]^ Data from the province of Ontario in Canada show that the prevalence of RA increased from 0.49% in 1996 to 0.9% in 2010.^[5]^ In Latin America, prevalence has been reported to be 2.8% in Mexico^[6]^ and 0.9% in Colombia, where 267,628 persons were estimated to be diagnosed with RA in 2005.^[7]^

Current treatments for RA include anti-tumor necrosis factor (TNF) alpha drugs, which have proven able to control disease progression and reduce the rate of disability.^[8]^ However, a high percentage of patients do not respond sufficiently well to this type of treatment.^[9]^ Given that therapy is expensive and can cause adverse events, factors that can predict an adequate response to treatment with anti-TNF alpha agents have been investigated.^[10,11]^ Rheumatoid factor (RF) is an autoantibody that binds to the Fc portion of human IgG. RFs are frequently detected in patients with RA or other autoimmune diseases but are also observed in patients with non-rheumatic conditions and even in healthy subjects.^[12]^ The indisputable role of RF and anti-cyclic citrullinated peptide antibody (ACPA) in the diagnosis of RA^[13]^ has led to studies in various populations aimed at determining the predictive ability of RF and ACPA assays with respect to prognosis.^[14,15]^ Some authors suggest that positive RF and ACPA titers can predict an inadequate response to therapy with anti-TNF alpha agents,^[16–19]^ whereas others report inconsistent and contradictory conclusions.^[20–22]^ Given the magnitude of the problem and the need for real-world evidence that will enable us to investigate this phenomenon in a patient population under standard conditions, the present study aims to establish whether RF or ACPA can predict remission or severe disability in patients with RA receiving treatment with anti-TNF alpha agents.

## 2 Methods

### 2.1 Design, population, and sample

An observational cohort study was performed, at a reference center for patients with RA in the city of Bogotá, Colombia, where care follows a treat-to-target strategy.^[23,24]^ The study center has a registry of all patients attended. Currently the cohort of RA has 3000 patients, and were included all patients aged ≥18 years with a confirmed diagnosis of RA according to the criteria of the American College of Rheumatology/EULAR,^[13]^ with an active disease in spite of had been under treatment with conventional disease-modifying antirheumatic drugs (DMARDs), and for whom a treatment scheme was begun with anti-TNF alpha medication (etanercept, infliximab, adalimumab, or golimumab), with a minimum follow-up time of 12 months. We excluded patients who did not have RF or ACPA at diagnosis. We also excluded patients with a health assessment questionnaire (HAQ) score >2 at admission in order to determine with certainty the incidence of disability at the end of follow-up.

### 2.2 Procedure for linking, follow-up, and data processing

The cohort database was created using the hospital information system based on the selection criteria described above. In order to guarantee data confidentiality, no names or surnames were used when the central database was queried. Each patient was assigned a consecutive identifier by order of inclusion in the working database. Queries made in SQL and data were subsequently imported as a plain text file to Excel. The source database was considered the master database and stored in the server of our institution. A copy of the master database was made for data processing and reclassification of variables of interest. Once the appropriate adjustments were made, the database was exported to Stata 14, which was used for the study analyses.

### 2.3 Variables of interest

The independent variables were RF and ACPA measured on a continuous numerical scale and classified as positive or negative, with cut-offs of ≥15 IU/mL for RF and ≥20 IU/mL for ACPA. When the 2 markers were positive (RF and ACPA), a co-positivity status was defined. The measurable clinical outcomes of interest were the disease activity score (DAS28) and HAQ scores at admission and periodically thereafter based on the criteria of the attending physician. The DAS28 was measured on a continuous scale and classified into subgroups in order to define remission, that is, a score of ≤2.6 for at least 3 months. The HAQ was analyzed on a continuous scale and classified into subgroups to define severe disability (≥2). The change in DAS28 was calculated as the difference between the baseline value and the value recorded at the last clinical follow-up visit. The same procedure was applied with the baseline and final HAQ data. Time to event was the interval between the admission date and the last effective follow-up visit or the date when the outcomes of interest were recorded (severe disability and remission). The variables age, sex, and treatment were also included in the analysis.

### 2.4 Statistical analysis

Descriptive analyses were made for each of the variables, and the most appropriate summary statistics were used. In the case of categorical variables, the data were summarized using frequency tables with absolute and relative values. In the case of the continuous variables, measures of central tendency and dispersion were used taking into account the distribution of the data.

Three independent variables were assessed. The first was RF, a continuous numerical variable, for which data were summarized using measures of central tendency and dispersion. Taking into account the cut-off point for positivity (≥15 IU/mL), we divided patients into 2 subgroups, namely, RF-positive and RF-negative. Subgroup data were expressed using absolute and relative frequencies.

The second independent variable was ACPA, whose value was reported on a continuous numerical scale and which was used for the descriptive analyses with measures of central tendency and dispersion. Taking into account the cut-off point for positivity (≥20 IU/mL), we divided patients into 2 subgroups, namely, ACPA-positive and ACPA-negative (<20 IU/mL). Subgroups data were expressed using absolute and relative frequencies. In addition, a third independent variable was the status of co-positivity, in patients with a positive result in both markers (RF and ACPA). The analysis for this predictor used the same pattern (absolute frequencies and percentages).

In order to evaluate the possible effect of the independent variables, we selected 2 measurable clinical variables for remission or disease activity (DAS28) and the degree of disability caused (HAQ). The result of the DAS28 was registered as a continuous numerical variable whose behavior was summarized using measures of central tendency and dispersion. The same patterns of analysis were applied in the case of the HAQ. Both DAS28 and HAQ were categorized, as described in the section on variables of interest.

Based on the results of the numerical scale, we calculated the differences reached between the baseline and final DAS28 score and thus obtained the change for each patient. We then investigated differences in the distributions of the positive and negative groups, both with RF (dichotomous variable) and ACPA (dichotomous variable). The Wilcoxon test was applied since the data were not normally distributed. The same procedure was used for HAQ.

Given the dynamic cohort design and taking into account the sum of times per person between inclusion in the cohort and outcome, we calculated the incidence rates (IRs) for remission and for severe disability in positive and negative patients. Based on these results, we obtained the incidence rate ratio (IRR), with its respective 95% confidence interval. This estimation was adjusted for possible confounders using the Poisson regression method.

Finally, using the Kaplan–Meier method, we calculated the probability of remission between 2 and 6 years by comparing the curves for RF-positive and RF-negative patients. The statistical comparison was made using the log-rank test. In all cases, the hypothesis was tested with a *P* value of <.05. Statistical analysis was performed in Stata 15.

### 2.5 Ethics

This study adheres to the principles of the Declaration of Helsinki (2013) and Resolution 8430 of 1993 of the Colombian Health Ministry. The information was guaranteed to be used only for scientific purposes, and the right to privacy was protected by omitting data that could identify the study participants. Given that the study center has an institutional registry for research purposes, the protocol required all patients to previously authorize the use of their clinical data for academic and research purposes by means of a document that is included in the clinical history. The protocol for this study was submitted to and approved by the Independent Ethics Committee of Hospital San José de Bogotá, Bogotá, Colombia.

## 3 Results

According to the inclusion criteria, 400 patients were included. The median age was 60 years, and 322 patients were women (80.5%). The median time of disease duration was 7 years. One hundred percent of patients had been treated with conventional DMARDs at usual doses (methotrexate until 20 mg per week, sulphasalazine until 3 g per day, chloroquine 250 mg per day, leflunomide 10 to 20 mg per day, and prednisolone until 7.5 mg per day). RF and ACPA were positive in 357 patients (89%) and 348 patients (87%), respectively. Co-positivity was present in 324 patients (simultaneous positive result- RF and ACPA: 81%). Follow-up ranged from 12 to 79 months, with a median of 41 months. Table 1 shows the baseline characteristics of the cohort, including level of disease activity and disability at admission. The data are shown according to whether patients had positive or negative results, both for RF and ACPA.

**Table 1:**
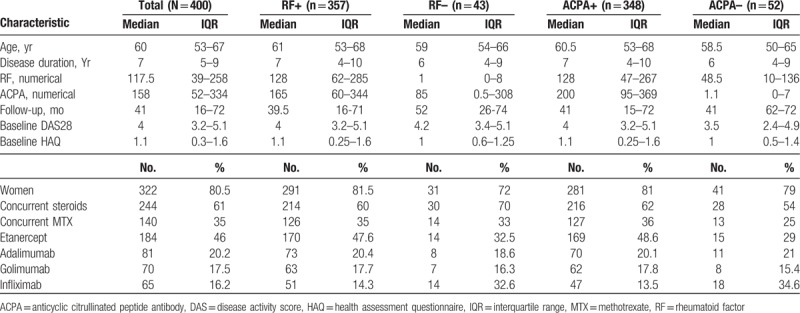
Baseline characteristics of the cohort.

### 3.1 Distribution of DAS28 and HAQ by subgroup

The distribution of the baseline DAS28 and baseline HAQ did not differ significantly between RF-positive and RF-negative patients. Similar behavior was observed for ACPA-positive and ACPA-negative cases (Table 2).

**Table 2:**
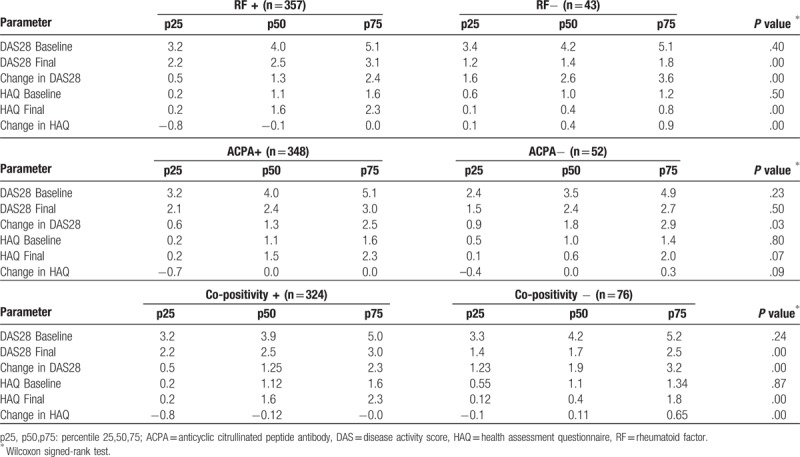
Change in DAS28-HAQ at baseline and end of study.

The median for the final measurement on the DAS28 was 2.5 for the RF-positive group, 1.4 for the RF-negative group. The median difference between the baseline and final DAS28 was 1.26 for RF-positive patients and 2.6 for RF-negative patients. The final measurement in the HAQ was 1.6 for RF-positive patients, compared with 0.4 for RF-negative patients. The change in HAQ was 0.37 in RF-negative patients and −0.12 in RF-positive patients. The results are shown in Table 2, as is the comparison with ACPA-positive and -negative patients, and the status of co-positivity.

### 3.2 Remission: IRs

There were 246 cases of remission during follow-up. A total of 1433 person-years of follow-up were recorded, with a remission rate of 17 cases per 100 person-years (95% CI, 15–19). The rate was 16.3 cases per 100 person-years (95% CI, 14–18) for RF-positive patients and 23.5 cases per 100 person-years for RF-negative patients (95% CI, 19–27). The remission rate was 16.7 (95% CI, 15–19) in ACPA-positive patients and 19.7 (95% CI, 15–25) in ACPA-negative patients. Table 3 shows the crude and adjusted IRRs with their respective confidence intervals.

**Table 3:**
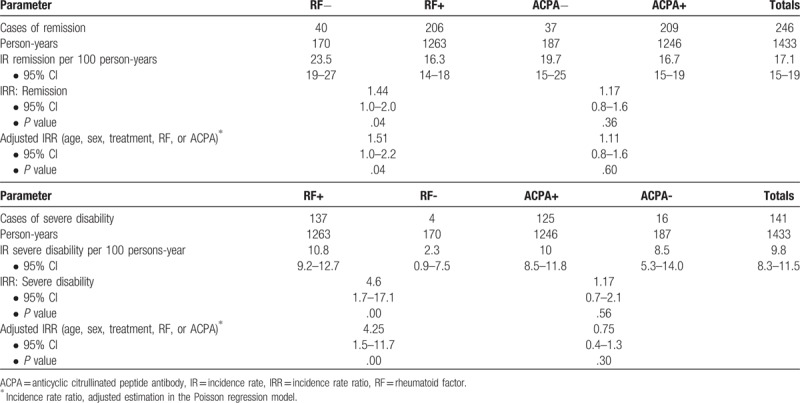
Incidence rate ratios for remission and severe disability.

The remission IR in the group of co-positivity was 15.9 cases per 100 person-years (95% CI, 13.8–18.4), and 22 cases per 100 person-years for the group of patients without this condition (95% CI, 17–28).

**Figure 1:**
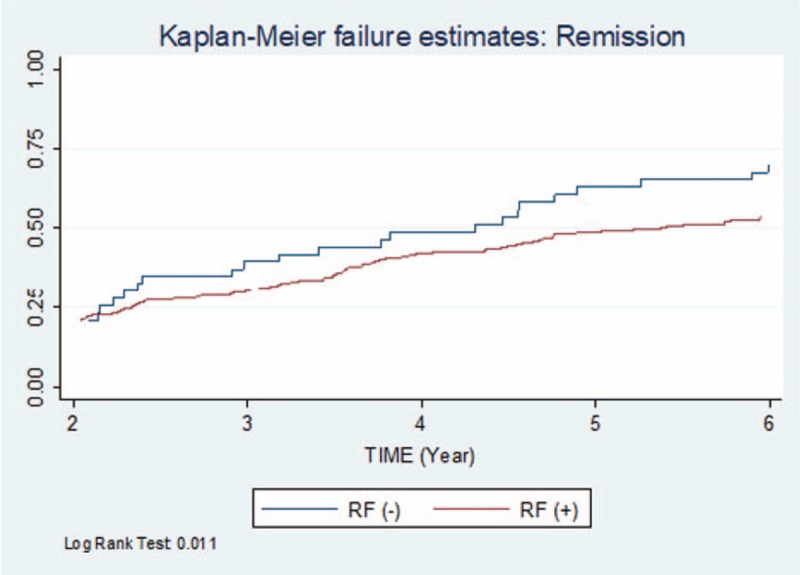
Kaplan–Meier failure estimates: remission. RF indicates rheumatoid factor.

The Kaplan–Meier curves comparing the function obtained for remission in the RF-positive and RF-negative cohorts revealed statistically significant results (*P *= .011 [log-rank]). Figure 1 shows the comparison of the function for each group.

### 3.3 Severe disability: IRs

There were 141 new cases of severe disability during follow-up, with an IR of 9.8 cases per 100 person-years (95% CI, 8.3–11.5). The incidence rate was 10.8 cases per 100 person-years (95% CI, 9.2–12.7) for RF-positive patients and 2.3 cases per 100 person-years (95% CI, 0.9–7.5) for RF-negative patients. The severe disability rate was 10 (95% CI, 8.5–11.8) in ACPA-positive patients, compared with 8.5 in the ACPA-negative group (95% CI, 5.3–14). Table 3 shows the crude and adjusted IRRs with their respective confidence intervals.

The severe disability IR in the group of co-positivity was 9.9 cases per 100 person-years (95% CI, 7.8–12.1), and 1.9 cases per 100 person-years for the group of patients without this condition (95% CI, 1.6–2.2).

**Figure 2:**
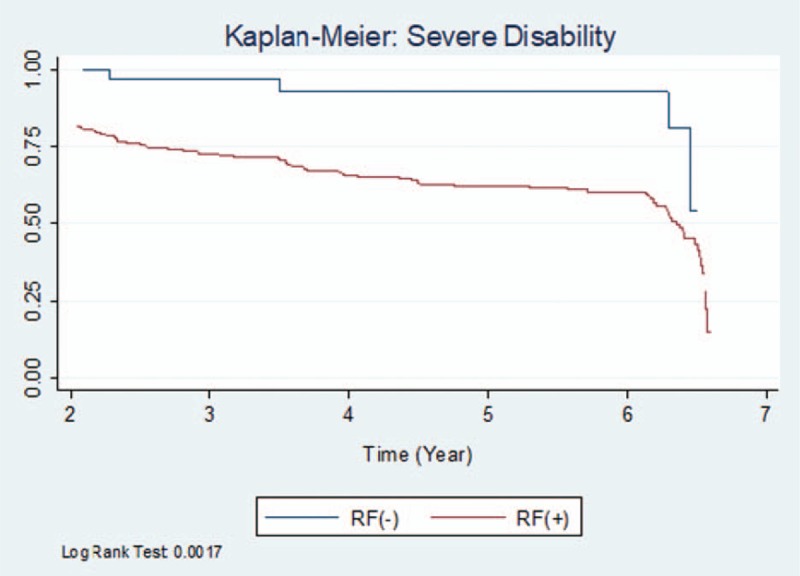
Kaplan–Meier: severe disability. RF indicates rheumatoid factor.

The comparison of survival functions (Kaplan–Meier) revealed significant differences for severe disability between the RF-positive and RF-negative cohorts (*P *= .0017 [log-rank]; Fig. 2).

## 4 Discussion

Various authors throughout the world have proposed that RF and ACPA are markers of the severity of RA, increased radiographic progression and joint damage.^[25–27]^ However, in the context of treatment with anti-TNF alpha drugs, it is essential to determine factors that enable us to predict prognosis. The present study was performed in the framework of an integrated care program run according to the premises of treat-to-target and adhering to the recommendations of centers of excellence in the treatment of RA.^[24]^ Data from our cohort (400 Colombian patients diagnosed with RA and receiving treatment with anti-TNF alpha drugs) enabled us to establish that a positive RF titer could predict a lower rate of remission and severe disability. This conclusion is based on a comparison of rates of remission and severe disability in RF-positive and RF-negative patients. Furthermore, despite differences in the distribution of changes in DAS28 between patients with positive and negative ACPA titers, significant differences were not found for rates of remission and severe disability between the 2 groups. Therefore, our results were not conclusive with respect to the predictive ability of ACPA. When analyzing the group of patients with RF and ACPA co-positivity, the results are consistent with the RF behavior analyzed individually. This allows us to infer that the most relevant factor in the co-positivity would be the RF.

Similarly designed studies report results that are consistent with ours. Such is the case of Potter et al,^[19]^ whose multicenter observational study in the United Kingdom reported clinical data for a cohort of 642 patients treated with 1 of 3 anti-TNF alpha drugs (infliximab, 46%; etanercept, 43%; and adalimumab, 11%). Of these patients, 89% and 82% had positive RF and ACPA, respectively. The study structure and methodological design are similar to ours, and the percentages of positivity for RF and ACPA are close to those we report. The authors evaluated the change in DAS28 after 6 months of treatment and found an average decrease of 2.5 points. Results for RF-positive and RF-negative patients were compared using a linear regression model in which the average decrease in DAS28 in the RF-negative group was 0.48 points higher than the average decrease in RF-positive patients. Our findings were similar, although our follow-up time was longer (median 41 months, range 12–79 months) and the decrease in DAS28 was 2.6 in the RF-negative patients and 1.26 in the RF-positive group, ie, a difference of 1.34 points between the groups.

As for the findings reported for ACPA, Potter et al^[19]^ found that the average decrease in DAS28 was 0.39 points greater in the ACPA-negative cohort than in the ACPA-positive cohort. This result is consistent with our finding that the mean decrease in DAS28 was 0.46 points higher in the ACPA-negative cohort.

Another observational study with a similar methodology was performed in Sweden, where a cohort of 689 patients with RA was followed for 5 years and remission (DAS28 <2.6) was assessed.^[28]^ The authors found that the probability of achieving this outcome in RF-positive patients was almost half that of RF-negative patients (OR, 0.55; 95% CI, 0.38–0.85). This result was evaluated for ACPA, and the authors found an association at 18 months of follow-up, although the result was not consistent at 60 months. These findings are very consistent with ours, given that the rate of remission we report for RF-negative patients was 51% higher than that of RF-negative patients (*P *= .04). The result is comparable with those for ACPA, where remission rates were similar for both ACPA-positive and ACPA-negative patients (IRR, 1.1; 95% CI, 0.76–1.63).

A key area of discussion in the present study is the role of observational studies based on institutional registries in daily clinical practice. The subject has been addressed by other authors, who concluded that so-called real-world studies are essential for determining aspects such as effectiveness and safety of therapeutic alternatives (e.g., anti-TNF alpha agents).^[29,30]^ Aaltonen et al^[29]^ evaluated the effectiveness of anti-TNF alpha agents under real-world conditions and determined the percentage of patients who were eligible for the clinical trials performed. Based on data from the Finnish national register for biologic treatment in rheumatic diseases, they found that 47% of treated patients achieved remission and that only 7.6% to 44% of registered cases would have been eligible to participate in a clinical trial. The authors concluded that the results obtained in the different clinical trials cannot necessarily be extrapolated to the routine care of patients with RA and proposed the presence of discrepancies between the results of studies that evaluated the efficacy of anti-TNF alpha drugs^[31,32]^ and those of registry-based real-world studies.^[30,33]^ The hypothesis underlying these differences would be that clinical trial outcomes are evaluated in highly selected populations, thus leading to an overestimation of the effect and underestimation of possible adverse events. Therefore, clinical research should be complemented with studies that provide real-world evidence, such as ours, where previous findings are confirmed and other results are tested.

Based on these arguments, Mancarella et al^[30]^ performed a multicenter observational study in 14 reference centers for rheumatic diseases in Italy. The population included 1257 patients with active RA treated with anti-TNF alpha agents. After 6 months of follow-up, the authors showed that 24% of patients in the RF-positive group had achieved DAS28 <2.6, whereas in the RF-negative group, this percentage was 36%, that is, a relative risk of 1.5, which is similar to the IRR reported in our study (adjusted IRR, 1.51; 95% CI, 1.05–2.18). As for level of disability, the authors compared percentages for patients who, after 6 months of follow-up, were classified as having low-level disability, and found that the percentage was lower in the RF-positive group than in the RF-negative group (45% vs 58%). Our findings are consistent with these, although it is worth mentioning that the outcome we proposed was the opposite (severe disability) and our follow-up time was longer. Thus, the severe disability rate was almost 5-fold greater in RF-positive patients than in RF-negative patients.

With respect to control of possible confounders, we stress findings such as the rate of remission among men, which, according to our results, was 39% higher than in women (IRR, 1.39; 95% CI, 1.05–1.88), as reported by Forslind et al,^[28]^ who found that after a 2-year follow-up, only 32% of women had achieved remission, compared with 48% of men (*P* = .001). Therefore, it is important to note that the estimations presented in our study were adjusted for possible confounders such as sex, age, and treatment with biologics.

The prognosis and clinical course of patients with RA have changed thanks to the effectiveness of biologics, including anti-TNF alpha agents. These have played an essential role, given their demonstrated effectiveness profile, within a safety framework outweighed by the expected benefit.^[8,34,35]^ This observation is confirmed by our results: the cohort of patients receiving therapy with anti-TNF alpha agents achieved a remission rate of 17 cases per 100 person-years. However, the comparison of remission rates between RF-positive and RF-negative patients, in addition to being statistically significant, is clinically relevant and must play a role in managing the aspirations of the patient and the treating clinicians. It also paves the way for drugs that enable higher remission rates in patients who continue to have positive marker titers.

It is worth highlighting that our results are based on a minimum follow-up of 12 months, although in some patients this reached 6 years. It is also important to highlight that remission was only taken into account when the patient reached DAS28 <2.6 points and maintained it for the last 3 months of follow-up. Nevertheless, given the importance of the results obtained, we intend to perform a prospective cohort study with a larger study population and longer follow-up so that we can evaluate the consistency of our findings and refine what might be considered potential weaknesses of our study.

RF is an example of a preexisting antibody binding the Fc fragment of IgG1 which is the IgG subtype used to engineer the majority of anti-TNF complete monoclonal antibodies. RF is commonly of the IgM class, although IgG and IgA RFs have been described. Since IgM is a pentamer and therefore decavalent, it can bind up to 10 IgG antibodies per molecule of RF, leading to the formation of large immune complexes.^[36]^ Anti-TNF-formed immune complexes can be eliminated by endocytosis, thus reducing their bioavailability and half-life and facilitating complement activation.^[37,38]^

In summary, the present study enabled us to show that the remission rate for RF-negative patients was higher than that of RF-positive patients. We also showed that the rate of severe disability was higher in RF-positive patients than in RF-negative patients. It is important to continue investigating the role of the level of RF at the beginning of the disease, because the blockade of therapeutic target and control strategies in RA may thus differ in patients in relation with baseline RF titers, and may not solely be based on RF-positivity or negativity state at baseline. Finally, it is worth mentioning that our findings were not conclusive for ACPA.

## Author contributions

**Conceptualization:** Pedro Santos-Moreno, Guillermo Sánchez, Carlos Castro.

**Data curation:** Guillermo Sánchez, Carlos Castro.

**Formal analysis:** Guillermo Sánchez.

**Funding acquisition:** Pedro Santos-Moreno, Carlos Castro.

**Investigation:** Pedro Santos-Moreno, Guillermo Sánchez.

**Methodology:** Guillermo Sánchez, Carlos Castro.

**Project administration:** Guillermo Sánchez.

**Software:** Guillermo Sánchez.

**Supervision:** Pedro Santos-Moreno.

**Validation:** Pedro Santos-Moreno.

**Writing – original draft:** Pedro Santos-Moreno, Guillermo Sánchez.

**Writing – review & editing:** Pedro Santos-Moreno, Guillermo Sánchez, Carlos Castro.

Guillermo Sánchez orcid: 0000-0002-4954-7803.

## References

[1] Ramírez LA Rodríguez C Cardiel MH Burden of illness of rheumatoid arthritis in Latin America: a regional perspective. Clin Rheumatol 2015;34 Suppl 1:S9–15. 26219487 10.1007/s10067-015-3012-0 PMC4617840

[2] Lee DM Weinblatt ME Rheumatoid arthritis. Lancet 2001;358:903–11. 11567728 10.1016/S0140-6736(01)06075-5

[3] Boonen A Severens JL The burden of illness of rheumatoid arthritis. Clin Rheumatol 2011;30 suppl 1:S3–8. 21359507 10.1007/s10067-010-1634-9

[4] Gabriel SE Michaud K Epidemiological studies in incidence, prevalence, mortality, and comorbidity of the rheumatic diseases. Arthritis Res Ther 2009;11:229–45. 19519924 10.1186/ar2669 PMC2714099

[5] Widdifield J Paterson JM Bernatsky S The epidemiology of rheumatoid arthritis in Ontario, Canada. Arthritis Rheumatol 2014;66:786–93. 24757131 10.1002/art.38306

[6] Peláez-Ballestas I Sanin LH Moreno-Montoya J Epidemiology of the rheumatic diseases in Mexico. a study of 5 regions based on the COPCORD methodology. J Rheumatol Suppl 2011;86:3–8. 21196592 10.3899/jrheum.100951

[7] Díaz-Rojas JA Dávila-Ramírez FA Quintana-López G Rheumatoid arthritis prevalence in Colombia: an approach based on burden of disease study during 2005. Revista Colombia Reumatol (Engl Ed) 2016;23:11–6.

[8] Santos-Moreno P Sanchez G Gomez D Direct comparative effectiveness among 3 anti-tumor necrosis factor biologics in a real-life cohort of patients with rheumatoid arthritis. J Clin Rheumatol 2016;22:57–62. 26886438 10.1097/RHU.0000000000000358 PMC4927323

[9] Lv Q Yin Y Li X The status of rheumatoid factor and anti-cyclic citrullinated peptide antibody are not associated with the effect of anti-TNFalpha agent treatment in patients with rheumatoid arthritis: a meta-analysis. PLoS One 2014;9:1–0. 10.1371/journal.pone.0089442 PMC3937352 24586782

[10] Gibbons LJ Hyrich KL Biologic therapy for rheumatoid arthritis: clinical efficacy and predictors of response. BioDrugs 2009;23:111–24. 19489652 10.2165/00063030-200923020-00004

[11] Kristensen LE Kapetanovic MC Gülfe A Predictors of response to anti-TNF therapy according to ACR and EULAR criteria in patients with established RA: results from the South Swedish Arthritis Treatment Group Register. Rheumatology (Oxford) 2008;47:495–9. 18316338 10.1093/rheumatology/ken002

[12] Ingegnoli F Castelli R Gualtierotti R Rheumatoid factors: clinical applications. Dis Markers 2013;35:727–34. 24324289 10.1155/2013/726598 PMC3845430

[13] Aletaha D Neogi T Silman AJ 2010 Rheumatoid arthritis classification criteria: an American College of Rheumatology/European League Against Rheumatism collaborative initiative. Arthritis Rheum 2010;62:2569–81. 20872595 10.1002/art.27584

[14] Taylor P Gartemann J Hsieh J A systematic review of serum biomarkers anti-cyclic citrullinated peptide and rheumatoid factor as tests for rheumatoid arthritis. Autoimmune Dis 2011;2011:1–8. 10.4061/2011/815038 PMC3170888 21915375

[15] Katchamart W Johnson S Lin HJ Predictors for remission in rheumatoid arthritis patients: a systematic review. Arthritis Care Res (Hoboken) 2010;62:1128–43. 20235210 10.1002/acr.20188

[16] Canhao H Rodrigues AM Mourao AF Comparative effectiveness and predictors of response to tumour necrosis factor inhibitor therapies in rheumatoid arthritis. Rheumatology (Oxford) 2012;51:2020–6. 22843791 10.1093/rheumatology/kes184 PMC3475979

[17] Cuchacovich M Catalan D Wainstein E Basal anti-cyclic citrullinated peptide (anti-CCP) antibody levels and a decrease in anti-CCP titres are associated with clinical response to adalimumab in rheumatoid arthritis. Clin Exp Rheumatol 2008;26:1067–73. 19210871

[18] Tanaka Y Takeuchi T Inoue E Retrospective clinical study on the notable efficacy and related factors of infliximab therapy in a rheumatoid arthritis management group in Japan: one-year clinical outcomes (RECONFIRM-2). Mod Rheumatol 2008;18:146–52. 18283523 10.1007/s10165-008-0026-3 PMC2279153

[19] Potter C Hyrich KL Tracey A Association of rheumatoid factor and anti-cyclic citrullinated peptide positivity, but not carriage of shared epitope or PTPN22 susceptibility variants, with anti-tumour necrosis factor response in rheumatoid arthritis. Ann Rheum Dis 2009;68:69–74. 18375541 10.1136/ard.2007.084715 PMC2596303

[20] Bobbio-Pallavicini F Caporali R Alpini C High IgA rheumatoid factor levels are associated with poor clinical response to tumour necrosis factor alpha inhibitors in rheumatoid arthritis. Ann Rheum Dis 2007;66:302–7. 17079248 10.1136/ard.2006.060608 PMC1856018

[21] Lequerré T Jouen F Brazier M Autoantibodies, metalloproteinases and bone markers in rheumatoid arthritis patients are unable to predict their responses to infliximab. Rheumatology (Oxford) 2007;46:446–53. 16899502 10.1093/rheumatology/kel262

[22] Soto L Sabugo F Catalan D The presence of anti-citrullinated protein antibodies (ACPA) does not affect the clinical response to adalimumab in a group of RA patients with the tumor necrosis factor (TNF) (-308 G/G promoter polymorphism. Clin Rheumatol 2011;30:391–5. 21234628 10.1007/s10067-011-1679-4

[23] Smolen JS Aletaha D Bijlsma JW Treating rheumatoid arthritis to target: recommendations of an international task force. Ann Rheum Dis 2010;69:631–7. 20215140 10.1136/ard.2009.123919 PMC3015099

[24] Santos-Moreno P Castaneda O Garro B From the model of integral attention to the creation of centers of excellence in rheumatoid arthritis. Clin Rheumatol 2015;34 Suppl 1:S71–77. 26208443 10.1007/s10067-015-3017-8 PMC4617853

[25] Agrawal S Misra R Aggarwal A Autoantibodies in rheumatoid arthritis: association with severity of disease in established RA. Clin Rheumatol 2007;26:201–4. 16572283 10.1007/s10067-006-0275-5

[26] Ates A Kinikli G Turgay M Effects of rheumatoid factor isotypes on disease activity and severity in patients with rheumatoid arthritis: a comparative study. Clin Rheumatol 2007;26:538–45. 16804738 10.1007/s10067-006-0343-x

[27] Berglin E Johansson T Sundin U Radiological outcome in rheumatoid arthritis is predicted by presence of antibodies against cyclic citrullinated peptide before and at disease onset, and by IgA-RF at disease onset. Ann Rheum Dis 2006;65:453–8. 16176994 10.1136/ard.2005.041376 PMC1798112

[28] Forslind K Hafström I Ahlmén M Sex: a major predictor of remission in early rheumatoid arthritis. Ann Rheum Dis 2007;66:46–52. 17158139 10.1136/ard.2006.056937 PMC1798403

[29] Aaltonen KJ Ylikyla S Tuulikki Joensuu J Efficacy and effectiveness of tumour necrosis factor inhibitors in the treatment of rheumatoid arthritis in randomized controlled trials and routine clinical practice. Rheumatology (Oxford) 2017;56:725–35. 28064209 10.1093/rheumatology/kew467

[30] Mancarella L Bobbio-Pallavicini F Ceccarelli F Good clinical response, remission, and predictors of remission in rheumatoid arthritis patients treated with tumor necrosis factor-alpha blockers: the GISEA study. J Rheumatol 2007;34:1670–3. 17611987

[31] Goekoop-Ruiterman YP de Vries-Bouwstra JK Allaart CF Clinical and radiographic outcomes of four different treatment strategies in patients with early rheumatoid arthritis (the BeSt study): a randomized, controlled trial. Arthritis Rheum 2005;52:3381–90. 16258899 10.1002/art.21405

[32] van der Heijde D Klareskog L Rodriguez-Valverde V Comparison of etanercept and methotrexate, alone and combined, in the treatment of rheumatoid arthritis: two-year clinical and radiographic results from the TEMPO study, a double-blind, randomized trial. Arthritis Rheum 2006;54:1063–74. 16572441 10.1002/art.21655

[33] Hyrich KL Watson KD Silman AJ Predictors of response to anti-TNF-alpha therapy among patients with rheumatoid arthritis: results from the British Society for Rheumatology Biologics Register. Rheumatology (Oxford) 2006;45:1558–65. 16705046 10.1093/rheumatology/kel149

[34] Flouri I Markatseli TE Voulgari PV Comparative effectiveness and survival of infliximab, adalimumab, and etanercept for rheumatoid arthritis patients in the Hellenic Registry of Biologics: Low rates of remission and 5-year drug survival. Semin Arthritis Rheum 2014;43:447–57. 24012040 10.1016/j.semarthrit.2013.07.011

[35] Ruiz Garcia V Burls A Cabello JB Certolizumab pegol (CDP870) for rheumatoid arthritis in adults. Cochrane Database Syst Rev 2017;9:1–300. 10.1002/14651858.CD007649.pub4 PMC6483724 28884785

[36] van Schie KA Wolbink GJ Rispens T Cross-reactive and pre-existing antibodies to therapeutic antibodies—effects on treatment and immunogenicity. MAbs 2015;7:662–71. 25962087 10.1080/19420862.2015.1048411 PMC4623040

[37] Deng R Loyet KM Lien S Pharmacokinetics of humanized monoclonal anti-tumor necrosis factor-{alpha} antibody and its neonatal Fc receptor variants in mice and cynomolgus monkeys. Drug Metab Dispos 2010;38:600–5. 20071453 10.1124/dmd.109.031310

[38] Gorevic PD Rheumatoid factor, complement, and mixed cryoglobulinemia. Clin Dev Immunol 2012;2012:439018. 22956968 10.1155/2012/439018 PMC3432568

